# Supplementary material for: TargetDB: A target information aggregation tool and tractability predictor
Source: PLoS One. 2020 Sep 2;15(9):e0232644. doi: 10.1371/journal.pone.0232644 (PMC7467329; doi:10.1371/journal.pone.0232644)
Supplement: S1 File — Details of the different methods, datasources and calculations performed for the creation of TargetDB, discussion on the machine learning model. (DOCX) [file pone.0232644.s001.docx]

**TargetDB: A target information aggregation tool and tractability predictor**

Stephane P. De Cesco^1,*^, John B. Davis^1^ and Paul E. Brennan^1,^*

^1^ ARUK Oxford Drug Discovery Institute, Target Discovery Institute, Nuffield Department of Medicine, University of Oxford, NDMRB, Roosevelt Drive, Oxford, Oxfordshire, OX3 7FZ, United-Kingdom

* Corresponding authors

E-mail: [paul.brennan@ndm.ox.ac.uk](mailto:paul.brennan@ndm.ox.ac.uk) (PEB), [sdecesco@gmail.com](mailto:sdecesco@gmail.com) (SPD)

SUPPORTING INFORMATION

**Table A. Datasources used.**

| **ChEMBL** | SQLite database download (Version 25) | [CC BY-SA 3.0](http://creativecommons.org/licenses/by-sa/3.0/) |
| --- | --- | --- |
| **DrugEBIlity** | File from ftp://ftp.ebi.ac.uk/pub/databases/chembl/DrugEBIlity/ | ? |
| **BindingDB** | Files from https://www.bindingdb.org/bind/chemsearch/marvin/SDFdownload.jsp | [CC BY 3.0 US](http://creativecommons.org/licenses/by/3.0/us/) |
| **TCRD** | SQL database download from http://juniper.health.unm.edu/tcrd/download/ | TCRD is made available under the CC-BY-SA 4.0 license. For details, see https://creativecommons.org/licenses/by/4.0/ Please note that TCRD incorporates many data sources with a variety of associated licenses. Details of the TCRD source data are located here: http://juniper.health.unm.edu/tcrd/download/TCRD_DataSourceLicenses.xslx |
| **PDB** | Files downloaded from http://files.rcsb.org/download/ | From https://www.rcsb.org/pages/policies "free of all copyright restrictions and made fully and freely available for both non-commercial and commercial use. Users of the data should attribute the original authors of that structural data" |
| **PDBBind** | File download from http://www.pdbbind.org.cn/download.asp | License agreement available there: http://www.pdbbind.org.cn/enroll.asp |
| **Humanmine** | Python API | LGPL license |
| **OpenTargets** | Python API | Open source and open access |
| **Human Protein Atlas** | Files downloaded | [CC BY-SA 3.0](http://creativecommons.org/licenses/by-sa/3.0/) |
| **HGNC** | Files from https://www.genenames.org/download/statistics-and-files/ | Publicly available data |
| **PubMed** | Via Eutils API | ? |
| **UniProt** | Web API at http://www.uniprot.org/uniprot/ | [CC BY-ND 3.0](http://creativecommons.org/licenses/by-nd/3.0/) |

# Category scoring

Each category is scored according to different pieces of information that are weighted according to their importance. Here below each category score will be detailed. If not specified, the global score is the average of the individual scores and are always between 0 and 1.

**Biology**

- Protein expression levels (bio_EScore = 1 if available)
- Number of antibodies (bio_AScore = 1 if count >50)
- Variants (bio_VScore = 1 if count >0)
- Mutants (bio_MScore = 1 if count >0)
- Mice genotypes (bio_GScore = 1 if count >0)
- KEGG/Reactome (bio_PScore = 1 if KEGG+Reactome data =0.5 if KEGG or Reactome = 0 if none available)

**Chemistry**

**Height (Scale from 0 to 1)**

- BindingDB potent cpds (Score = log(count), normalized (0->1))
  - Compounds with activity < 100 nM
- ChEMBL potent cpds (Score = log(count), normalized (0->1))
  - Compounds with activity < 100 nM

**Color ( low= red // high = green )**

- Score = 1
  - Count of BindingDB ligands that are labelled phase 2 > 0
- Score = 0.8
  - Count of ChEMBL ligands with great selectivity > 0
- Score = 0.7
  - Count of ChEMBL ligands with good selectivity > 0
- Score = 0.6
  - Count of ChEMBL ligands with moderate selectivity > 0
- Score = 0.3
  - Count of potent ChEMBL ligands > 0

OR

- - Count of potent BindingDB ligands > 0
- Score = 0
  - All of the above not met

**Structure**

**Height (Scale from 0 to 1)**

- % of sequence covered
  - Percent of the sequence covered by PDB structures
- % of domains covered
  - Percentage of the domains that are covered by PDB structures
- Number of PDB
  - if count >=1 🡺 Score = 0.25
  - if count >=2 🡺 Score = 0.5
  - if count >=3 🡺 Score = 1
- Number of alternate (BLAST) PDB
  - Same as number of PDB * max similarity

**Color ( low= red // high = green )**

- Pockets druggability scores
  - - Average of pockets with a druggability score > 0.5
  - Domain druggability
    - Average of domains druggability and tractability coming from DrugEBIlity
  - Alternate (BLAST) pockets druggability scores
    - Average of pockets with a druggability score > 0.5 * max similarity

**Disease link**

- Number of disease area (OpenTargets)
  - Score = 0.5 if count = 1 / Score = 1 if count > 1
- Max association score (OT)
  - OpenTargets max association score
- Diseases count unitprot
  - Score = 1 if any
- Diseases count tcrd
  - Score = 1 if any

**Disease global score** = (Max association score * 2 + Number of diseases area + Diseases count uniprot + diseases count tcrd) / 5

**Genetic link**

**Height (Scale from 0 to 1)**

- Log_10_(gwas count*10) capped at 2, normalized (0->1)
- OpenTargets MAX genetic association score * normalisation depending on the number of associations with this maximum score
  - Normalisation factor
    - Log_2_(count of association with max score) capped at 5 normalized (0->1)

**Color ( low= red // high = green )**

- Count of significant gwas associations (p-value <= 5e-9) / total count of gwas associations
- Avg score for the top10 open-targets associations

**Information**

- Log(JensenLab Pubmed score) capped at 12 then normalized (0->1)

**Safety**

**Height (Scale from 0 to 1 = availability of data)**

- Log(Number of genotypes) capped at 6, normalized (0->1) + 0.3 if expression data available, capped to 1

**Color ( potential safety liability = red // safe = green )**

- Genotypes (safe_GScore)
  - - 2 * Count of homozygote genotype with lethal phenotype + count of heterozygote genotype with lethal phenotype – count of heterozygote genotype with normal phenotype – 2 * count of homozygote genotype with normal phenotype
- Expression profile (safe_EScore)
  - - Heart protein expression

$$T= Kd for different targets$$

- - - Liver protein expression
    - Kidney protein expression
      - If any of the above is high or 1stddev higher than all tissue average 🡺 score = 1

$$\rho_{i}=Probability of a Kd value$$

- - - - Else 🡺 score = 0

# Calculation of selectivity metrics

The Shannon entropy was used as a measure of selectivity for the two following metrics. The global equation is the following:

$$S_{sel}=-\sum_{i}^{T} \rho_{i} \log\rho_{i}$$

With *S_sel_* = Selectivity Entropy

**Selectivity of compound**

T = K_d_ for different targets

$\rho_{i}$ = Probability for a K_d_ value

$$\rho_{\left( T \right)}=\frac{{K_{d}}_{T}}{\sum_{i} {K_{d}}_{i}}$$

Where K_dT_ = Binding association for target T

**Selectivity of tissue expression**

T = Expression in different tissue

$\rho_{i}$ = Probability of an expression value

$$\rho_{(T)}=\frac{E_{T}}{\sum_{i} {E_{T}}_{i}}$$

Where E_T_ = Expression value in tissue T

# Multi-Parameter Optimisation (MPO) score

In order to help the ranking of a large list of targets for prioritization purpose, an MPO score was constructed. While a simple weighted average of all the individual categories scores would provide an overall view of the amount of information available on a given target, it would not necessarily mean it is ideal for target prioritization. Moreover, not everyone might have the same criteria/requirements for target selection. One might prioritize targets with chemical information while someone else might deprioritize such targets and favour genetic association data.
To achieve that, we developed a scoring system where every category is associated with a coefficient that the user can define. When the list mode is selected, a dialog box opens asking the user to choose a number between 200 and -200 for each category. The default value is 100, it represents the weighted average if all categories are set to 100. Setting a value higher than that will prioritize targets with a high score in that category, setting a value of 0 will ignore that category in the MPO score and negative values will deprioritize targets with a high score and prioritize targets with a low score in that category. This approach allows the user to tune the prioritization parameters for their own criteria. The exact formula for the MPO calculation is described hereunder:

| $CScoreS=\left( CScore-0.5 \right)*2$ | $\left\{ -1\to1 \right\}$ |
| --- | --- |
| $W=\frac{Weights}{100}$ | $\left\{ -2\to2 \right\}$ |
| $MPO Score= \frac{\sum_{i} {CScoreS}_{i}*W_{i}}{\sum_{i} \left\vert W_{i} \right\vert}$ | $\left\{ -1\to1 \right\}$ |
| $MPO Score \left( scaled \right)= \frac{MPO Score}{2}+0.5$ | $\left\{ 0\to1 \right\}$ |

# Tractability model generation

In order to further assist the decision-making process, we decided to evaluate the possibility to generate a model that would output both a YES/NO answer for tractability as well as a probability of tractability. To do so, the individual component of the score above were used as features and evaluated in different machine learning models using the scikit-learn package in python. Below is the process that led to the model. You will also find the jupyther notebook file as part of the supporting information (S5 File).

For building this classification model, it is essential to provide to the model a group of validated tractable targets as well as a group of intractable targets. While the first one is easily manageable, thanks to the vast amount of literature defining tractable targets, it is far more challenging to come up with a list of intractable targets. Therefore, we decided to simply select randomly 400 targets from a list of targets from which all the targets in the druggable genome list were removed. That left us with a list of 13500 targets to select from. On the other hand, the tractable targets were selected from the clinically actionable list from DGIdb (n=399). Once combined, they were then separated into two groups: Training set (n=560) and the Test set (n = 240). Each group containing an equal ratio of tractable/intractable targets (ratio = 0.5). The complete list of targets as well as their assignement in training or testing set is available in S4 File.


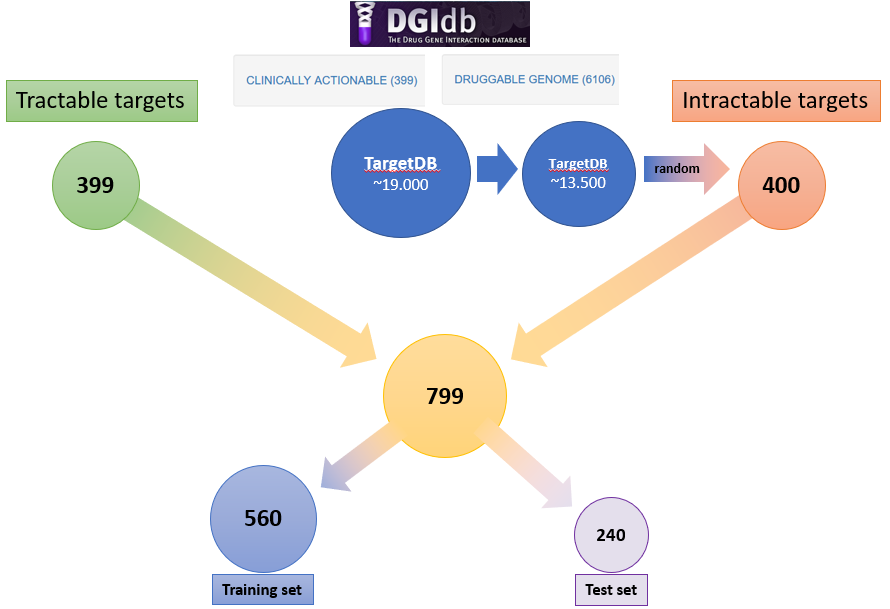


**Fig A. Machine learning data**

The training set was then used to assess different machine learning algorithm in order to select the best performing one. In order to assess them, different metrics where used:

$\boldsymbol{Accuracy}$ $\frac{TP+TN}{\left( TP+TN+FP+FN \right)}$

$\boldsymbol{Precision}$ $\frac{TP}{TP+FP}$

$\boldsymbol{Recall-}TP rate$ $\frac{TP}{TP+FN}$

$\boldsymbol{Fall}\text{-}\boldsymbol{out-}FP rate$ $\frac{FP}{FP+TN}$

$\boldsymbol{F}\boldsymbol{1-score}$ $2* \frac{1}{\frac{1}{precision} + \frac{1}{recall}}$

$\boldsymbol{AU-ROC}$ $Area under curved when the TP rate and FP rate$

$$are plotted at different threshold values$$

$TP=True positive TN=True negative$

$$FP=False positive FN=False negative$$

Before building the models, a correlation matrix was generated for the features in order to remove overlapping features. Based on this several colinear features were removed prior to ML models screening.


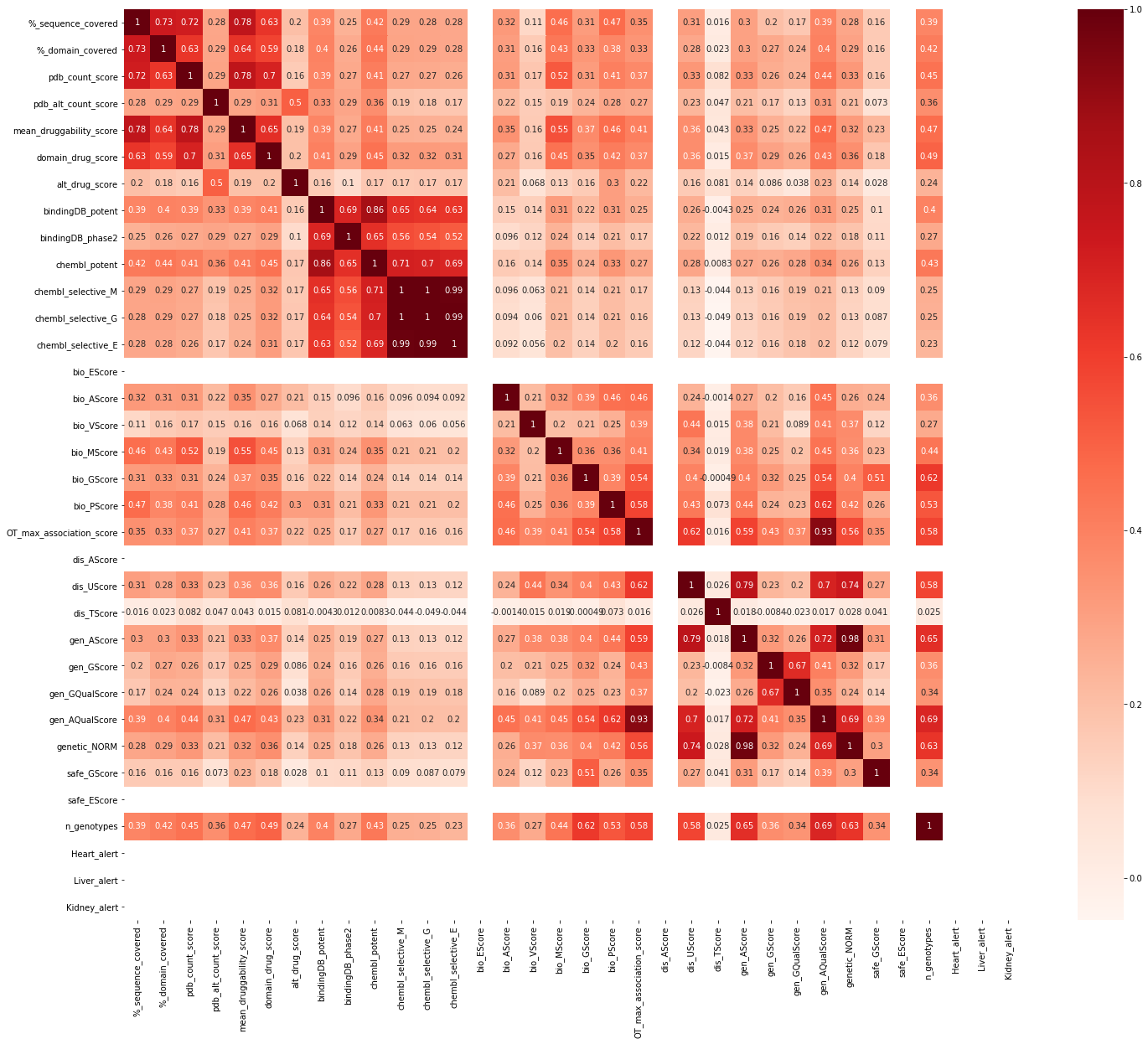


**Fig B. Correlation matrix of the features explored**

Several models were tried: steepest gradient descent (sgd), random forest, support vector machine (SVM), Gaussian process, k-neighbors, Naïve Bayes, Quadratic discriminant analysis (QDA) and Adaptive Boosting (AdaBoost). As a result of this first screening, the random forest model was picked as it results with the most balanced outcome between precision, recall, and accuracy, yielding the best F1-score from all the model. It is to be noted that these results were obtained using the cross-validation method with 5 subdivisions.

**Table B. Performance metrics for different algorithm tested**


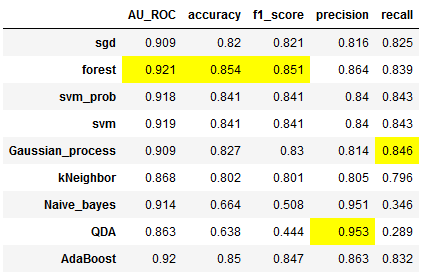


Before further optimizing the random forest model, we decided to look at the importance of each feature and see if the available features can be further reduced or if some features are too prominent in the prediction.


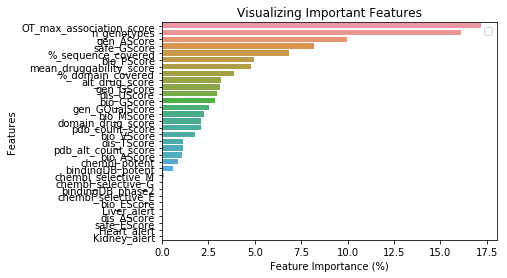


**Fig C. Features importance.** Feature importance for the unoptimize model

Looking at the importance of the features it was decided to remove the OT_max_association_score (which is the maximum association score provided by OpenTargets) as the genetic association score (gen_AScore) is a composite of this feature. It is also interesting to note that the score associated with the number of compounds in phase 2 in BindingDB (BindingDB_phase2) is of no relevance in the model. All the features contributing little to the model were also removed. By removing these features and retraining the model, the importance of the features looks more balanced with only the genetic association and the number of mouse genotypes occupying a prominent role, which is acceptable as it has been shown that genetically validated targets improve the probability of favourable outcome of a drug discovery program.[1]


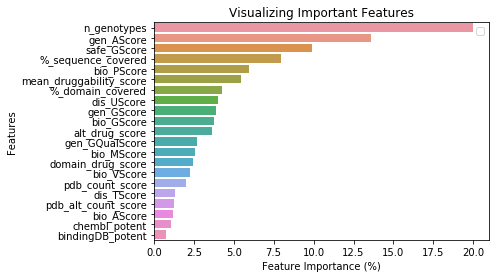


**Fig D. Feature importance of the refined model**

With these parameters, a series of optimization of the model parameters didn’t lead to any major improvement in the F1-score. It was then decided to continue with the default parameters.

As a final step, the test set was used against the model to control if the model was not overfitting the training set data and therefore be inapplicable to new data.

**Table C. Performance comparison.** Comparison of different parameters set for the random forest algorithm


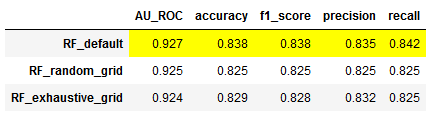


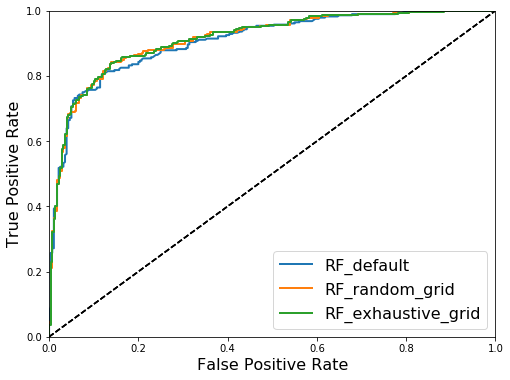


**Fig E. ROC curves.** Curves obtained for the different models of random forests

As you can see here, the default random forest model provides the best results with an F1-score that is in the same area than the one obtained on the training set (0.85 on the training set) further validating the model as a good predictor of tractability. The AU-ROC curves of the model provide a good evaluation of the performance of the model on the test set.

# Installation procedure:

1. Installing a python distribution

Requirement: Python version >=3.4 (For specific package dependencies please refer to the github page)

Suggested distribution: Anaconda 3 (<https://www.anaconda.com/distribution/>)

1. Install TargetDB python package

Preferred method: using pip package manager. Simply type in the terminal prompt:

pip install targetdb

1. Downloading and unpacking TargetDB Database

The pre-filled database can be download here : <https://github.com/sdecesco/targetDB/releases/download/v1.3.1/TargetDB_20_12_19.db.zip>

After download, simply unzip the file into your preferred folder (Unzipped file is large, >7Gb)

1. Launching TargetDB for the first time

Type “targetDB” in the terminal prompt and wait for a window to appear. The first time it is launched a configuration window will appear, prompting you to enter some informations


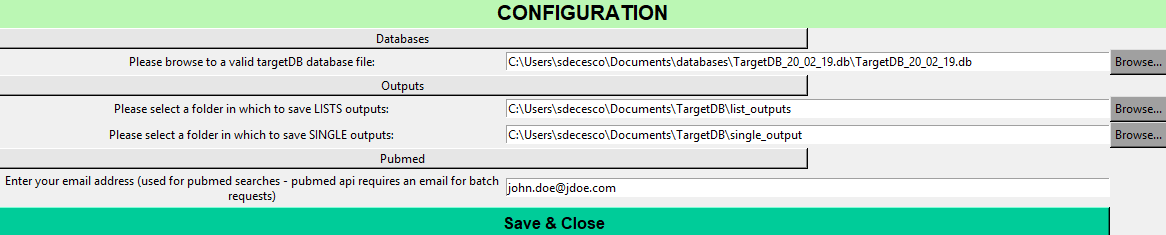


- - **Database** line: Tell the software where the database file was saved (see step 3)
  - **Outputs** (The program generates two types of outputs depending the mode used)
    - LISTS: Please indicate a folder in which it will save the lists outputs
    - SINGLE: Please indicate a folder in which it will save single outputs
      - *Note: These two folder can be the same*
  - **Pubmed:** TargetDB use a pubmed search to pull out relevant papers on targets or paper numbers if in list mode, it requires the user email address to use this functionality.

Once saved and closed the main TargetDB window will appear:


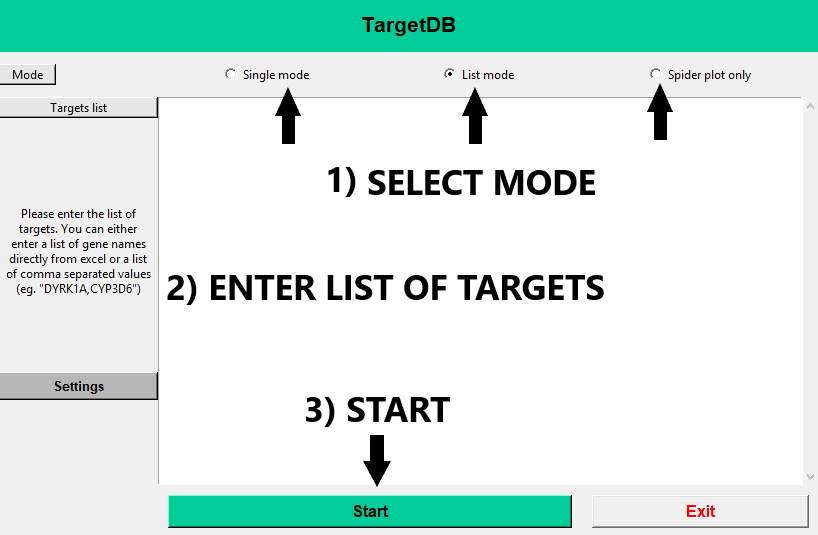


1. King EA, Davis JW, Degner JF. Are drug targets with genetic support twice as likely to be approved? Revised estimates of the impact of genetic support for drug mechanisms on the probability of drug approval. Marchini J, editor. PLOS Genet. 2019;15: e1008489. doi:10.1371/journal.pgen.1008489
