# Supplementary figures and images for: TargetDB: A target information aggregation tool and tractability predictor
Source: PLoS One. 2020 Sep 2;15(9):e0232644. doi: 10.1371/journal.pone.0232644 (PMC7467329; doi:10.1371/journal.pone.0232644)

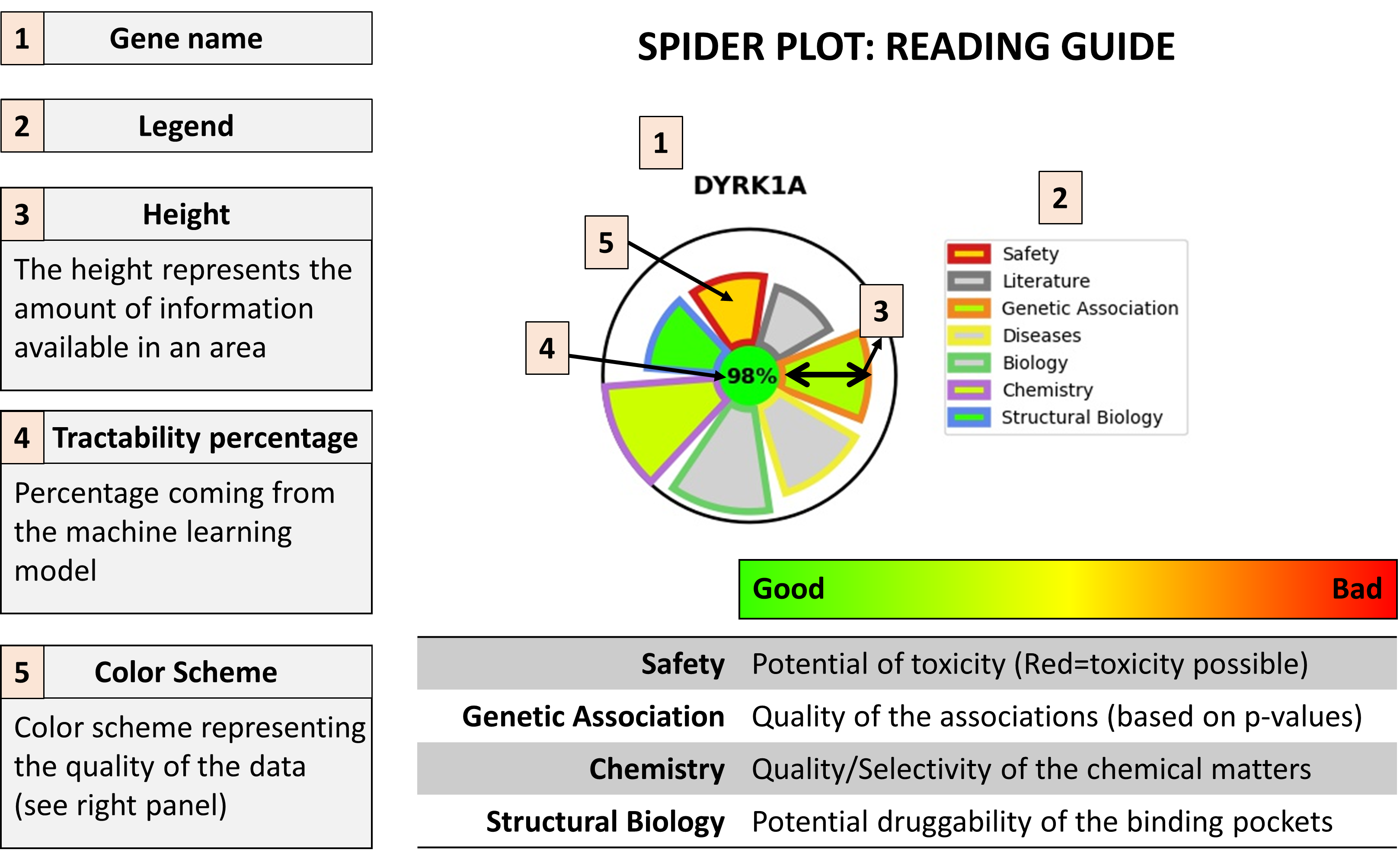

Supplement: S1 Fig — (PNG) [file pone.0232644.s009.png]
